# Suicidal ideation and suicide attempts among asthma

**DOI:** 10.1186/s12991-016-0122-2

**Published:** 2016-11-29

**Authors:** Jae Ho Chung, Sun- Hyun Kim, Yong Won Lee

**Affiliations:** 1 Department of Internal Medicine, International St. Mary’s Hospital, Catholic Kwandong University College of Medicine, Incheon, Republic of Korea; 2 Department of Family Medicine, International St. Mary’s Hospital, Catholic Kwandong University College of Medicine, Simgokro 100 Gil 25 Seo-gu, Incheon, 22711 Republic of Korea

**Keywords:** Suicidal ideation, Suicidal attempt, Asthma

## Abstract

**Background:**

The present study aimed to investigate the mental health status in patients with asthma and assess the effects of asthma on suicidal ideation and attempts using a representative sample from Korea.

**Methods:**

Individual-level data were obtained from 228,744 participants (6372 with asthma and 222,372 without asthma) of the 2013 Korean Community Health Survey. Demographic characteristics, socioeconomic status, physical health status, and mental health status were compared between patients with asthma and population without asthma. Multivariable logistic regression was performed to investigate the independent effects of the asthma on suicidal ideation and attempts.

**Results:**

A depressed mood for 2 or more continuous weeks was reported by 12.0% of subjects with asthma and 5.7% of controls (*p* < 0.001). Suicidal thoughts were reported by 21.4% of patients with asthma and 9.8% of controls (*p* < 0.001). Suicidal attempts were reported by 1.0% of the patients with asthma and 0.4% of controls (*p* < 0.001). Following adjustment for age, sex, income, education, job, marital status, smoking, alcohol, exercise, and presence of diabetes mellitus, hypertension, stroke, arthritis, and depression, the ORs for suicidal ideation with asthma were 1.53 (95% CI 1.42–1.65) and that for suicidal attempts was 1.32 (95% CI 1.01–1.73).

**Conclusions:**

We found that asthma increased the risk for suicidal ideation and attempts, even controlling for the effects of socioeconomic status, physical health status, comorbid chronic medical diseases, and depressive mood. Our finding suggests that asthma per se may be an independent risk factor for suicidality.

## Background

Many chronic diseases are complicated by emotional and psychological disorders and yet the emotional dimensions of such chronic diseases are frequently overlooked when medical treatment is considered [1]. Several studies have found statistically significant correlates between asthma and suicide ideation and suicide attempts [2–6].

Suicide is the fourth leading cause of death in Korea [7]. Suicide rate was 33.3 per 100,000 persons in Korea in 2011, which ranked first among the Organization for Economic Co-operation and Development (OECD) countries (https://data.oecd.org/healthstat/suicide-rates.htm). Since asthma and suicide are important public health burdens, epidemiological study investigating the mental health status in asthma patients and its relationship with suicidal ideations and attempts would be required to assess the potential risk for suicide, and ultimately prevent suicidal completion in patients with asthma. However, very few studies about the relationship between asthma and suicide have been done in Asia especially South Korea. There are a number of epidemiological differences among the studies that have evaluated suicidal behavior, and an investigation of the various comorbidities and risk factors that lead to suicide in different ethnic groups is therefore necessary. As far as I know, this is the first study suicidal prevalence among asthma patients in Korea.

We aimed to compare the status of mental health including suicidal ideations and attempts between patients with asthma and population without asthma using data from nationwide population-based health survey (The 2013 Korean Community Health Survey).

## Methods

### Study participants

For this study, we obtained data from the 2013 Korean Community Health Survey (KCHS, https://chs.cdc.go.kr/chs/index.do), which was carried out by the Korea Centers for Disease Control and Prevention (KCDC). The KCHS is a nationwide cross-sectional health interview survey, annually conducted since 2008, to investigate the patterns of disease prevalence and morbidity as well as personal lifestyle and health-related behaviors in adults aged 19 years or older. The sample size for the KCHS is 900 subjects in each of 253 community units, including 16 metropolitan cities and provinces. The KCHS expects a total of 227,700 survey participants per year, but the actual number of respondents approximates 230,000. The KCHS has a two-stage sampling process. The first stage involves selection of a sample area (tong/ban/ri) as a primary sample unit according to the number of households in the area using a probability proportional to size sampling technique. In the second stage, sample households are selected in each sample area (tong/ban/ri) using systematic sampling methods. This process ensures that the sample units can be representative of the entire population [8]. For the sample to be statistically representative of the population, the data from the survey are weighted based on the sample design. The KCHS employs interviewers who were trained in computer-assisted personal interviewing techniques to collect information.

The institutional review board at the Korea Centers for Disease Control and Prevention approved the study protocol (2013-06EXP-01-3C), and all participants gave written informed consents.

In total, 228,781 individuals participated in the 2013 survey. This study was based on 228,744 participants, excluding 37 with insufficient data to confirm a doctor’s diagnosis with asthma. The final analysis identified 6372 asthma individuals who had been diagnosed by a doctor.

### Baseline physical health

Physical health can affect an individual’s mental health and future mortality risk [3]. Conditions comorbid that include hypertension, diabetes, stroke, and arthritis were investigated in this study. The number of comorbid conditions was also evaluated which were based on the answer “yes” to the question “Were you diagnosed with diseases by a physician?” to avoid bias generated by subjective assessment.

### Socioeconomic status

Indicators of socioeconomic status are associated with suicidal thoughts [9], and the present study therefore evaluated education, occupation, and household income. Self-reported smoking, alcohol intake, and physical activity were estimated from questionnaire responses, and household income was categorized according to quartiles of total income for each member in the household. Marital status was categorized as married, single, or divorced/separated/widowed.

Suicide-related thoughts and behaviors are associated with health behaviors such as cigarette smoking [10], alcohol consumption [11], and exercise [12]. Thus, the present study assessed health behaviors such as smoking, drinking, and regular exercise using self-reported questionnaires. People who had smoked 100 cigarettes (5 packs) or more in their lifetime and currently smoked were classified as a ‘smoker,’ while everyone else belonged to the ‘non-smoker’ group. Risky drinking was defined as drinking more than five alcoholic beverages on one occasion, and individuals who had drunk more than 12 drinks on one occasion during the previous year were classified as risky drinkers [13]. Regular exercise was defined as routine walking at least five times per week for at least 30 min at a time or engaging during the survey period in regular moderate (at least five times per week for at least 30 min at a time) or strenuous (at least three times per week for at least 20 min at a time) exercise as defined by the American College of Sports Medicine Guidelines [14]. Self-rated health status was analyzed using a 5-point scale, with responses of ‘very good’, ‘good’, ‘normal’, ‘poor’, and ‘very poor’.

### Mental health measures

Psychosocial factors can affect the relationship between suicidal ideation and mortality, and suicidal ideation may also be considered as an indicator of psychosocial factors. Mental health surveys that included the same questions as those in the KCHS were provided to all participants. Three dimensions of mental health were determined within the domains of health status and mental health, namely stress, depression, and suicidal ideations and attempts. Participants reported their level of stress as none, mild, moderate, or severe. Depression was screened using the Korean version of the World Health Organization (WHO) Composite International Diagnostic Interview-Short Form (CIDI-SF), which was validated as a cost-effective screening instrument that could be easily integrated into health surveys [15]. The WHO CIDI-SF includes questions such as “In your lifetime, have you ever had 2 weeks or more when nearly every day you felt sad, blue, or depressed?” assess depression, subjects answered “yes” or “no” to a question of whether they had experienced a depressive mood for 2 or more continuous weeks during the previous year. Suicidal ideation was assessed by participants’ positive answer to the question “In the last 12 months, did you think about committing suicide?” A “yes”’ or “no” response was also used to determine whether the subjects had suicidal ideations; if the subject answered “yes,” they were then asked about their suicide attempts, if any. This indicator is a well-documented predictor of suicide attempts that has been previously used in other surveys of adults [16].

### Ethical issues

The institutional review board at the Korea Centers for Disease Control and Prevention approved the study protocol (2013-06EXP-01-3C), and all participants gave written informed consents.

### Data analysis

Descriptive statistical methods were used to describe the basic characteristics of the study population; the numbers and percentages are reported for each variable. Student’s *t* test and Chi-square test were used to compare variables between patients with stroke and population without stroke. Multivariable logistic regression analysis was conducted to calculate the adjusted odds ratios (ORs) for suicidal behavior among the stroke patients; we included age, sex, income, education, job, marital status, smoking, alcohol, exercise, presence of diabetes mellitus, hypertension, stroke, arthritis, and depression. Results were expressed with a 95% confidence interval (CI). All data were analyzed using the Statistical Package for Social Sciences (Version 20.0; IBM, Armonk, New York).

## Results

The baseline characteristics of the study population (*n* = 228,744) are presented in Table 1. As compared to population without stroke (*n* = 224,175), patients with stroke (*n* = 6372) were older and had higher proportions of female, smoker, being alone (divorced/separated/widowed), hypertension, stroke, arthritis, and had lower level of education, lower level of income, lower proportions of alcohol drinking, regular exercise, and less having a job (all *p* < 0.001).

**Table 1:** Clinical characteristics of study populations

	No asthma (*n* = 222,372)	Asthma (*n* = 6372)	*P* value
Age (years)	51.8 ± 17.0	61.1 ± 17.8	<0.001
Sex (Male %)	100,089 (45.0)	2605 (40.9)	<0.001
Smoking status			<0.001
Smoker	81,882 (36.8)	2562 (40.2)	
Non-smoker	140,490 (63.2)	3810 (59.8)	
Alcohol drinking	59,896 (26.9)	1050 (16.5)	<0.001
Regular exercise	114,714 (51.6)	3047 (47.8)	<0.001
Marital status			<0.001
Married	152,061 (68.4)	3844 (60.4)	
Single	33,101 (14.9)	683 (10.7)	
Divorced/separated/widowed	37,072 (16.7)	1839 (28.9)	
Job	140,886 (63.4)	2914 (45.8)	<0.001
Family income			<0.001
Low	52,214 (24.3)	2705 (43.9)	
Moderate-low	43,285 (20.2)	1258 (20.6)	
Moderate-high	70,830 (33.0)	1303 (21.1)	
High	48,400 (22.5)	887 (14.4)	
Education			<0.001
≤Elementary	57,240 (25.8)	3067 (48.2)	
Middle school	25,346 (11.4)	811 (12.8)	
High school	64,235 (29.0)	1197 (18.8)	
≥College	75,165 (33.8)	1282 (20.2)	
DM	11,623 (5.2)	306 (4.8)	0.075
Hypertension	53,068 (23.9)	2523 (39.6)	<0.001
Stroke	4305 (1.9)	252 (4.0)	<0.001
Arthritis	29,761 (13.4)	1884 (29.6)	<0.001

Differences in mental health status between asthma patients and population without asthma were presented in Table 2. Asthma patients reported more moderate to severe stress (33.4%) and depressive mood (12.0%) as compared to population without asthma (25.4%, *p* < 0.001; 5.7%, *p* < 0.001). The rate of asthma patients who had suicidal ideations (21.4%) was higher than twice the rate in population without asthma (9.8%, *p* < 0.001). The rate of asthma patients who had suicidal attempts (1.0%) was higher than three times the rate in population without asthma (0.4%, *p* < 0.001).

**Table 2:** Mental health of asthma patients

	No asthma (*n* = 222,372)	Asthma (*n* = 6372)	*P* value
Stress			<0.001
Moderate to severe	56,507 (25.4)	2120 (33.4)	
None to mild	165,685 (74.6)	4236 (66.6)	
Perceived health status			<0.001
Very good	13,177 (5.9)	142 (2.2)	
Good	73,803 (33.2)	1016 (16.0)	
Moderate	89,422 (40.2)	2194 (34.5)	
Bad	35,534 (16.0)	2010 (31.6)	
Very bad	10,381 (4.7)	1006 (15.8)	
Experiences of depressive mood for 2 or more continuous weeks	12,602 (5.7)	764 (12.0)	<0.001
Suicidal thoughts during the previous year	21,804 (9.8)	1360 (21.4)	<0.001
Suicidal attempts during the previous year	929 (0.4)	64 (1.0)	<0.001

The ORs of suicidal ideation and attempts among the asthma patients in comparison to population without asthma was presented in Table 3. A multivariate analysis adjusting for age and sex (model 1) revealed that the ORs for suicidal ideations and attempts were 2.01 (95% CI 1.89–2.14) and 2.42 (95% CI 1.87–3.12), respectively. When additional adjustments were performed for socioeconomic factors (i.e., family income, education, job, and marital status; model 2), the ORs for suicidal ideations and attempts were 1.84 (95% CI 1.72–1.96) and 2.12 (95% CI 1.63–2.75), respectively. After additional adjustments for factors related to physical health (i.e., smoking, alcohol, exercise, diabetes, hypertension, stroke, and arthritis; model 3), the ORs for suicidal ideations and attempts were 1.71 (95% CI 1.59–1.82) and 1.79 (95% CI 1.37–2.33), respectively. In a final model adjusted for all of factors including depressive mood (model 4), the ORs for suicidal ideations and attempts were 1.53 (95% CI 1.42–1.65) and 1.32 (95% CI 1.01–1.73), respectively.

**Table 3:** Odds ratio (95% CI) suicidal ideation and suicidal attempts for asthma

	Suicidal ideation OR (95% CI)	Suicidal attempts OR (95% CI)
Model 1	2.01 (1.89–2.14)	2.42 (1.87–3.12)
Model 2	1.84 (1.72–1.96)	2.12 (1.63–2.75)
Model 3	1.71 (1.59–1.82)	1.79 (1.37–2.33)
Model 4	1.53 (1.42–1.65)	1.32 (1.01–1.73)

Adjusted for age, sex variable in model 1. Adjusted for age, sex, family income, education, job, marital status variables in model 2. Adjusted for age, sex, family income, education, job, marital status, smoking, alcohol, exercise, physician diagnosed diseases (diabetes, hypertension, arthritis, stroke) variables in model 3. Adjusted for age, sex, income, education, job, marital status, smoking, alcohol, exercise, physician diagnosed diseases, depression in model 4

## Discussion

Our study showed that asthma patients had more severe stress, depressive mood, suicidal ideations, and attempts than population without asthma. In addition, asthma was associated with an increase in the risk for suicidal ideations and attempts, even after adjusting for factors that are known to increase suicidality such as socioeconomic status, chronic medical diseases, and depressive symptoms.

Asthma is an important chronic condition that has previously been linked to a number of adverse outcomes including depression and risk-taking behavior. There is currently a body of research suggesting a link between suicidal behavior and asthma [2–6]. Clarke and colleagues [5] examined data on 5692 adults aged 18 and older participating in a United States nationwide health study showed that 12% of the participants had a history of asthma, 8.7% had experienced suicidal ideation, and 4.2% had suicidal attempts. Despite adjustments for smoking, concurrent mental conditions and demographic factors, a statistically significant association observed between asthma and suicide ideation and attempt. Goodwin and Eaton [2] reported a relationship between asthma and increased likelihood of suicidal ideation (OR 2.3; 95% CI 1.03–5.3) and suicidal attempts (OR 3.5; 95% CI 1.4–9.0). The same result was found in Puerto Rico [6]. An analysis of 6584 adults whose data were drawn from the Third National Health and Nutrition Examination Survey (NHANES III) also reported an association between current asthma and suicide ideation (Odds Ratio 1.77) and suicide attempt (OR 3.26), after adjusting for confounding factors such as mood disorder, poverty, smoking, and demographics. Our study showed suicidal ideation (OR 1.53; 95% CI 1.42–1.65) and suicidal attempts (OR 1.32; 95% CI 1.01–1.73).

This present study cannot explain the mechanisms of the association between asthma and suicidal ideation and suicidal attempts. An association between asthma morbidity, risk-taking behavior, and depression has been presented in previous research, although the reasons and direction of this association are not clear [17]. Asthma may be associated with mood change, anxiety, and some difficulties in daily living which may themselves feel hopelessness and consequently increased suicide risk [18]. Another possible mechanism for this association concerns effects of hypoxia [19], and it has been suggested that an association between high altitude and suicide may be accounted for by metabolic stress associated with hypoxia in individuals who have mood disorders. Recent research reports that suicide rates are elevated in those living at higher altitudes [8, 20], smokers [21, 22] and asthma [6, 23]. A possible mechanism that was proposed is metabolic stress associated with hypoxia. Young SN propose that low brain serotonin synthesis due to hypoxia could be a factor in the high suicide rates seen in people living at altitude, smokers and patients with chronic obstructive pulmonary disease (COPD) and asthma [24]. As pulmonary function decreases, and as the disease progresses, the risk of alveolar hypoxia and consequent hypoxemia increases [25]. Another potential cause of depression in asthma sufferers is the use of particular medications, including corticosteroid or montelukast sodium, which, while reducing the symptoms of asthma, have also been linked to mood disturbances similar to the symptoms of major depression [26, 27].

The strength of our study is that data were obtained from a nationwide population-based survey with a large sample size (*n* = 228,744) and the sampling methods representative of the general population. Moreover, the survey provided information about a number of factors that might be related to suicidality, such as socioeconomic variables and physical health as well as mental health measures, which allows us to assess the independent effects of stroke on suicidality using multiple statistical adjustments.

Our study has some limitations that should be addressed. First, because this is a cross-sectional study, temporal relationship and causality between asthma and suicidality could not be determined. Second, all data in this survey are based on self-reported questionnaires; therefore, the recall bias leading to the possibility of over- or under-reporting cannot be excluded. In addition, our study sample might be biased toward mild asthma patients who could complete the questionnaires. Third, we could not obtain detailed information about severity of asthma.

## Conclusion

In summary, we observed that asthma patients had more depressive mood and suicidal ideation and attempts than population without asthma using a large population-based survey. We also found that asthma increased the risk for suicidal ideation and attempts, independent of other factors that are known to be associated with suicidality, suggesting that stroke per se may be an independent risk factor for suicidality. Given that a previous suicidal attempt is among the strongest risk factors for future attempt [28], our findings may warrant the need for physicians to screen for suicidality and provide psychosocial support as well as interventions for preventing suicide in management of asthma patients. Hence any treatment modality for asthma, to minimize the possibility of suicide in patients with asthma, must incorporate. More research is required in the mental health field and asthma, but the indicators are clear that there is a significant association between asthma and suicide. Patients with asthma must be assessed not only for physical health but also for psychological morbidity.
