# Erratum to: Suicidal ideation and suicide attempts among asthma

**DOI:** 10.1186/s12991-017-0127-5

**Published:** 2017-02-20

**Authors:** Jae Ho Chung, Sun-Hyun Kim, Yong Won Lee

**Affiliations:** 1 Department of Internal Medicine, International St. Mary’s Hospital, Catholic Kwandong University College of Medicine, Incheon, Republic of Korea; 2 Department of Family Medicine, International St. Mary’s Hospital, Catholic Kwandong University College of Medicine, Simgokro 100 Gil 25 Seo-gu, Incheon, 22711 Republic of Korea

## Erratum to: Ann Gen Psychiatry (2016) 15:35 DOI 10.1186/s12991-016-0122-2

The article [1] has been updated. The previously published version contained some errors/mistakes. The word ‘stroke’ has been changed to ‘asthma’ in the following sections: (1) in Data Analysis, the word in the 5th, 6th and 8th lines of the first paragraph on page 3 has been modified; (2) in Results, the term in the 3rd and 4th lines of the first paragraph has been corrcted; (3) in Discussion, the word in the 9th line of the second paragraph on page 5 has been revised; (4) in Conclusion, the 8th line of the first paragraph on page 5 has been changed.
